# Low-energy structures of clusters supported on metal fcc(110) surfaces

**DOI:** 10.1186/1556-276X-6-633

**Published:** 2011-12-15

**Authors:** Peng Zhang, Liuxue Ma, Hezhu Shao, Jinhu Zhang, Wenxian Zhang, Xijing Ning, Jun Zhuang

**Affiliations:** 1 Key Laboratory of Micro and Nano Photonic Structures (Ministry of Education), Department of Optical Science and Engineering, Fudan University, Shanghai 200433, China; 2 Applied Ion Beam Physics Lab, Institute of Modern Physics, Fudan University, Shanghai 200433, China

**Keywords:** supported cluster, structure, shape, metal surface

## Abstract

The low-energy structures (LESs) of adatom clusters on a series of metal face-centered cubic (fcc) (110) surfaces are systematically studied by the genetic algorithm, and a simplified model based on the atomic interactions is developed to explain the LESs. Two different kinds of LES group mainly caused by the different next nearest-neighbor (NNN) adatom-adatom interaction are distinguished, although the NNN atomic interaction is much weaker than the nearest-neighbor interaction. For a repulsive NNN atomic interaction, only the linear chain is included in the LES group. However, for an attractive one, type of structure in the LES group is various and replace gradually one by one with cluster size increasing. Based on our model, we also predict the shape feature of the large cluster which is found to be related closely to the ratio of NN and NNN bond energies, and discuss the surface reconstruction in the view of atomic interaction. The results are in accordance with the experimental observations.

**PACS**: 68.43.Hn; 68.43.Fg.

## Introduction

In the next-generation microelectronics and ultra-high-density recording, the fully monodispersed nanostructures are believed to be one of the most promising materials [1]. In order to fabricate such nanostructures, knowledge of the morphology of nanoclusters on surfaces becomes enormously important. So far, numerous experimental observations and theoretical investigations into structures of clusters have been reported on transition and noble fcc metal surfaces, e.g., fcc(111), fcc(100), and fcc(110) surfaces [2-10]. However, such studies mainly focus on the lowest-energy structures. For the structures with energy close to the lowest one, which are named low-energy structures here, investigations and discussions are far from enough. At the usual experimental temperature, besides the lowest-energy structure, the low-energy ones also appear frequently owing to thermal effect and usually play significant role in many surface thermodynamic processes [11]. In earlier publications, the low-energy structures of adatom cluster on fcc(111) have been systematically studied, and it has been shown how the atomic interactions determine the equilibrium structures and shapes of the supported clusters [10]. In order to get a global view on the morphology of supported homoepitaxial clusters, here we investigate further a series of metal homoepitaxial clusters on fcc(110) surfaces, whose structure characteristics are far different from those of fcc(111).

## Calculation method

Four metal homoepitaxial systems are investigated: Ni, Cu, Pt, and Ag. The atomic interactions are described by semiempirical potentials. The semiempirical potential might not be as accurate as the first-principle method in describing atomic interaction, but it enables us to study systematically clusters in a large size range, which is quite expensive for the latter one. Considering of the shortcoming of the semiempirical method, here we focus on the relationship between the atomic interaction and the structure of cluster, which is not sensitive to the accuracy of potential. However, we still choose the potentials carefully that nicely describe the surface diffusion [12,13]. For Ni and Cu, the atomic interactions are described by the embedded-atom method (EAM) potential given by Oh and Johnson [14] and the potential developed by Rosato, Guillopé, and Legrand (RGL) on the basis of the second-moment approximation to the tight-binding model [15,16], respectively. While, for Pt and Ag, the atomic interactions are all modeled by the surface-embedded atom method (SEAM) potential given by Haftel and Rosen for the surface environment [17,18].

Clusters are put on a slab containing 12 atom layers in *Z *direction, in which three bottoms of them are fixed to simulate a semi-infinite slab, while the atom numbers in *X *and *Y *directions vary with the cluster size *n*. Periodic boundary conditions are applied in *X *and *Y *directions. The clusters with size *n *= 2 to 39 are studied. Structures are optimized according to their energy by the genetic algorithm (GA), which has been described in detail in our previous publications [7,8].

## Results and discussion

**Figure 1:**
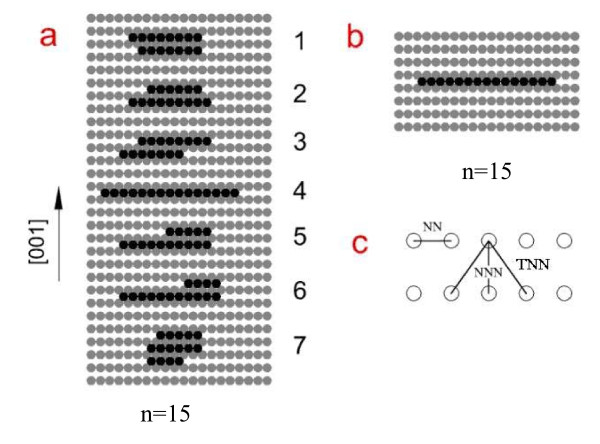
**Low-energy structures of cluster *n *= 15 (a) on Cu(110) and (b) on Pt(110) surface**. From structures 1 to 7, the energy is increasingly higher. The nearest-neighbor (NN), next nearest-neighbor (NNN), and third nearest-neighbor (TNN) bonds are indicated in (**c**).

In the present work, we investigate the structures whose energy differences with the lowest one are smaller than 0.12 eV. These structures are defined as the low-energy structures (LESs). According to the Boltzmann distribution, the probability of finding a structure whose energy is 0.12 eV higher than the lowest one is less than 1% at room temperature. Under the definition of LES above, we see that the low-energy structures obtained by our genetic algorithm are all two-dimensional on the surfaces studied here, i.e., three-dimensional structures are excluded from the LES group for their higher energy. However, on the different surface, the structure features are different as expected. On Ni(110), Ag(110), and Cu(110) surfaces, various types of structures are included in the LES group. In Figure 1, for example, the low-energy structures of cluster *n *= 15 obtained by our genetic algorithm on Cu(110) and Pt(110) are given. On Cu(110) surface, as shown in Figure 1a, both the linear chain and two-dimensional islands appear. The energy of linear chain is lower than that of three-row islands and higher than those of short two-row islands. While on Pt(110) surface, as shown in Figure 1b, there is only one structure type in the LES group, i.e., the linear chain along the [$1\bar{1}0$]. For other cluster sizes, the results are similar to those of cluster *n *= 15 on Ni(110), Ag(110), Cu(110), and Pt(110), i.e., the structure types of LES on Ni(110), Ag(110), and Cu(110) surfaces are various and change with the cluster size, while on Pt(110) surface, only one type of structure is included in LES group.

In order to understand the results on Ni(110), Ag(110), Cu(110), and Pt(110) surfaces, and give a general relationship between the structure and the atomic interaction, we try to give a simplified or approximated model in the following for describing the energy of the system, which is based on only the two-body atomic interaction. We decompose the total internal energy *E *of the system into three parts:

(1) $$E=E_{\text{aa}}+E_{\text{as}}+E_{\text{slab}},$$

where *E*_aa_, *E*_as_, and *E*_slab _refer to the energies contributed by the adatom-adatom interaction, the adatom-substrate interaction, and the bare slab internal interaction, respectively. For *E*_aa_, we consider the nearest-neighbor (NN), next nearest-neighbor (NNN), and third nearest-neighbor (TNN) interactions, and then *E*_aa _can be written as:

(2) $$E_{\text{aa}}=-\left(C_{\text{nn}}E_{\text{nn}}+C_{\text{nnn}}E_{\text{nnn}}+C_{\text{tnn}}E_{\text{tnn}}\right),$$

where *C*_nn_, *C*_nnn_, and *C*_tnn _refer to the numbers of NN, NNN, and TNN bonds, respectively. *E*_nn_, *E*_nnn_, and *E*_tnn _are the energies of NN, NNN, and TNN bonds, respectively. As shown in Figure 1c, one NNN bond generally corresponds to two TNN bonds, i.e., *C*_tnn _≈ 2*C*_nnn_. Therefore, the last two terms in the right side of Equation 2 can be written as *C*_nnn_*E*_nnn_+*C*_tnn_*E*_tnn_=*C*_nnn_(*E*_tnn+_2*E*_tnn_). For convenience, we set (*E*_nnn _+ 2*E*_tnn_) as $E_{\text{nnn}}^{*}$, i.e., $E_{\text{nnn}}^{\text{*}}=E_{\text{nnn}}+\text{2}E_{\text{tnn}}$. Considering the number of TNN bonds has fixed proportion with that of NNN bonds, and the TNN atomic interaction is much weaker than the NNN atomic interaction, we regard $E_{\text{nnn}}^{*}$ as the effective bond energy of NNN bond. Therefore, Equation 2 can be written as:

(3) $$E_{\text{aa}}=-\left(C_{\text{nn}}E_{\text{nn}}+C_{\text{nnn}}E_{\text{nnn}}^{\text{*}}\right).$$

The values of *E*_nn _and $E_{\text{nnn}}^{*}$ can be obtained by comparing the cohesive energies of structures with different *C*_nn _and *C*_nnn _[8]. For the adatom-substrate interaction, our calculation shows that it is not sensitive to the configuration of cluster and thus *E*_as _can approximately be viewed as a linear function of cluster size *n*, i.e., $E_{\text{as}}=-nE_{\text{as}}^{0}$, where $E_{\text{as}}^{0}$refers to the cohesive energy contributed by adatom-substrate interaction of one adatom. Then, Equation 1 can be written as:

(4) $$E=-\left(C_{\text{nn}}E_{\text{nn}}+\;C_{\text{nnn}}E_{\text{nnn}}^{*}\right)-nE_{\text{as}}^{0}+\;E_{\text{slab}}$$

Considering that the energy contributed by the bare slab internal interaction, i.e., *E*_slab_, can be approximately viewed as invariant, we then get the energy difference Δ*E *between the two structures as following:

(5) $$\Delta E=-\left(\Delta C_{\text{nn}}E_{\text{nn}}+\Delta C_{\text{nnn}}E_{\text{nnn}}^{\text{*}}\right).$$

Equation 5 shows that the energy difference of two structures results from the different nearest-neighbor and effective next nearest-neighbor adatom-adatom interactions.

By examining the structure feature of cluster on fcc(110) surface, one can see the numbers of NN and NNN bonds satisfy:

$$C_{\text{nn}}=n-r$$

(6) $$C_{\text{nnn}}=n-l,$$

in which *r *and *l *are the numbers of rows and lines of the cluster, respectively. For example, structure 1 in Figure 1a, r = 2 and *l *= 8, then *C*_nn _= 13 and *C*_nnn _= 7. With Equations 4 and 6, total internal energy can be written as:

$$E=-\left[\left(n-r\right)E_{\text{nn}}+\left(n-l\right)E_{\text{nnn}}^{*}\right]-nE_{\text{as}}^{0}+\;E_{\text{slab}}$$

(7) $$\;=\left[\left(r+l∕\xi \right)-\left(\text{1}+\text{1}∕\xi \right)n\right]E_{\text{nn}}-nE_{\text{as}}^{0}+\;E_{\text{slab}},$$

where $\xi =E_{\text{nn}}∕E_{\text{nnn}}^{*}$. In Equation 7, only one term $\left(r+l∕\xi \right)E_{\text{nn}}$is relevant to the structure. We denote $\left(r+l∕\xi \right)$ as structure factor *Φ*, i.e.,

(8) Φ=r+l∕ξ. 

With this simplified model Equation 7, for different structures of a cluster, we can predict their energy sequence just by comparing the values of *Φ*, which can be easily obtained by counting the numbers of rows and lines. Note that the bond energy *E*_nn _is always positive, the larger structure factor *Φ *then means the higher energy of the structure, and vice versa. In other words, the lowest-energy structure should have the smallest structure factor *Φ*.

**Figure 2:**
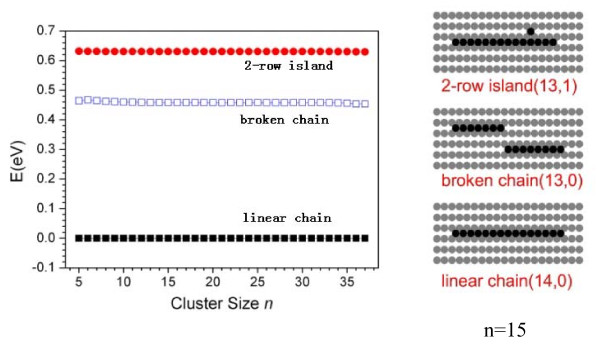
**The relative energy distribution. For simplification only the lowest energies of liner chain (solid squares), broken chain (open squares), and two-row island (dots) on Pt(110) surface are given**. The structures of cluster *n *= 15 as an example are shown in the right. The number in the bracket means the number of NN and NNN bonds, respectively.

On Pt(110) surface, our calculation shows that the bond energy $E_{\text{nnn}}^{*}$ is negative, which means the effective next nearest-neighbor adatom-adatom interaction is repulsive, and then the parameter $\xi =E_{nn}∕E_{nnn}^{*}<0$. According to Equation 8, when *r *is minimized and *l *is maximized, i.e., *r *= 1 and *l *= *n*, structure factor *Φ *reaches the minimum and the corresponding structure has the lowest energy. The *r *= 1 and *l *= *n *suggest that the cluster has the linear chain structure. Therefore, the linear chain is always the lowest-energy structure on Pt(110). In Figure 2, we give the relative energy distribution of linear chain, broken chain, and two-row island. For the broken chain and two-row island, only their lowest energies are shown for simplification. As shown in Figure 2, there are obvious gaps among the two-row island and linear and broken chains, and these gaps generally keep unchanged with cluster size increasing. For the broken chain, we have *r *= 2 and *l *= *n*, and for the two-row island, *r *= 2 and *l *≤ *n − *1. According to Equation 7, the energies of broken chain and two-row island are much higher than that of linear chain; the energy differences with linear chain are, respectively, *E*_nn _and ≥($E_{\text{nn}}-E_{\text{nnn}}^{*}$). Based on our calculation, both *E*_nn _and ($E_{\text{nn}}-E_{\text{nnn}}^{*}$) are much larger than 0.12 eV, the energy difference for defining the low-energy structure here. Therefore, both the two-row island and broken chain are always excluded from the LES group. As to islands with more than two rows, their energies are even much higher than that of two-row island because they have more NNN and less NN bonds, and they are also not included in the LES group. That is to say, the simplified model Equation 7 explains well the result of GA optimization on Pt(110) surface, where only one structure type, i.e., the linear chain appears in the LES group.

On Ni(110), Cu(110), and Ag(110) surfaces, different from the case on Pt(110), the calculation shows that the bond energy $E_{\text{nnn}}^{*}$ is positive. Then, $\xi =E_{\text{nn}}∕E_{\text{nnn}}^{*}>0$, which means, according to Equations 7 and 8, the structures with low energy on Ni(110), Cu(110), and Ag(110) surfaces should have proper numbers of rows and lines to ensure low structure factor *Φ*. For example, *n *= 15 on Cu(110) as shown in Figure 1a, the proper values include *r *= 2, *l *= 8; *r *= 1, *l *= 15; *r *= 3, *l *= 6, etc., because the structures with these values have low energy and all of them are included in the LES group. If the structures with the same row are classified as one structure type, then the LES group on Cu(110), also on Ni(110) and Ag(110) surfaces, contains several types of structures. When the cluster size increases, it is easy imaginable that the structure types will change for keeping the proper values of *r *and *l*. It is indeed true as shown in our GA optimization results and closely related to the type change of the lowest-energy structure, the details of which are described later.

In our previous work [8], we reported the type change of the lowest-energy structure at critical size $n=n_{R}^{c}$. Here, with the model Equation 7, we can further give the explicit expression for $n_{R}^{c}$. When the lowest-energy structure changes from *R *rows to *R *+ 1 rows, the number of lines will change from *L *to (*L *− *dl*) correspondingly, where *dl *is the decrement of number of lines. The change of the structure type at $n=n_{R}^{c}$, according to Equation 7, means that the configuration with (*R + *1) rows and (*L *− *dl*) lines instead of *R *rows and *L *lines has the lowest *Φ*. Namely *R*+*L*/*ξ*>*R*+1+(*L-dl*)/*ξ*, i.e., *dl*>*ξ*. The *dl *should be an integer, and then *dl*>*ξ *means

(9) dl=Int(ξ)+1

**Figure 3:**
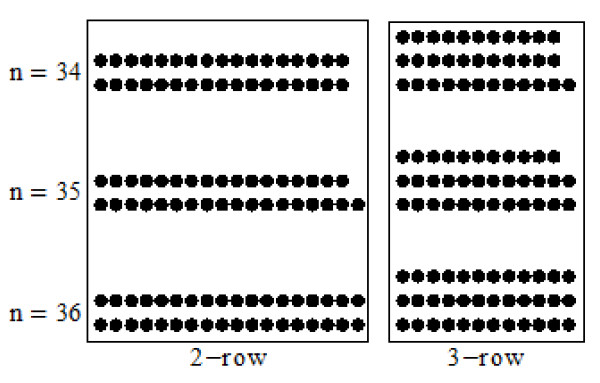
**The lowest-energy structures with two and three rows for cluster *n *= 34, 35 and 36, respectively**.

For example, on Cu(110) surface, our calculation gives the ratio of *E*_nn _and $E_{\text{nnn}}^{*}$, which is *ξ *= 5.26, and then *dl *= 6. In Figure 3, we give some structures of *R*-row and (*R *+ 1)-row types (*R = *2) whose energies are the lowest in their own type. At the critical cluster size $n_{R}^{c}$, *R*-row structure changing to (*R *+ 1)-row structure also means that their energy difference reaches minimum. According to our model Equation 7, in which only NN and NNN bonds are considered, the most probability for these two structures is that they are all perfect rectangle as shown in Figure 3 at size *n *= 36. Therefore, we have $n=RL=\left(R+1\right)\left(L-dl\right)=36$. Then whether *n *= *RL *= (*R*+1)(*L*-*dl*) is just the critical size $n_{R}^{c}$? We see cluster *n *= 35, at which Equation 9 is also satisfied, i.e., *dl *= *Int*(*ξ*)+1 = 6, and the relationship

(10) $$RL=\left(R+1\right)\left(L-dl\right)$$

is still valid. If we continue to subtract one atom, i.e., *n *= 34, the *R*-row structure with one-less line comparing with that of *n *= 36 appears, as shown in Figure 3, because such structure can keep the energy being lowest according to the model Equation 7. As a result, from *R*-row structure to *R *+ 1 one at *n *= 34, *dl = *5 no longer satisfies Equation 9. Therefore, on Cu(110) surface, the critical size for two-row type changing to three-row one should be $n_{R}^{c}=\text{35}$, and it can be written in general form,

$$n_{R}^{c}=RL-\left(R-1\right)$$

With Equations 9 and 10, we can finally fix the critical size, which satisfies:

(11) $$n_{R}^{c}=R\left(R+1\right)\;\left[Int\left(\xi \right)+1\right]-R+1$$

**Figure 4:**
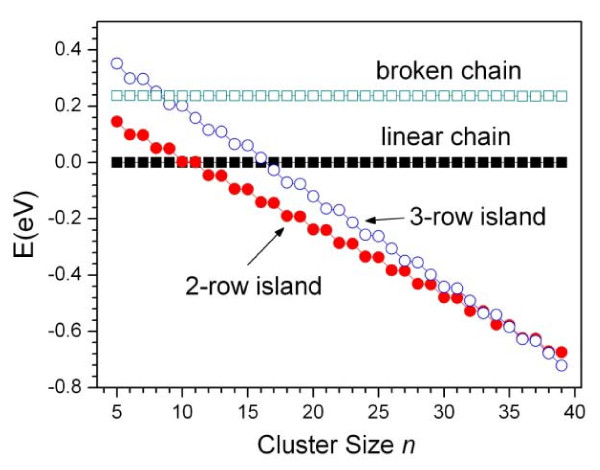
**The relative energy distribution**. The energies of liner chain (solid squares), broken chain (open squares), two-row islands (dots), and three-row islands (open circles) are given on Cu(110) surface. Only the lowest energies are considered as before.

**Figure 5:**
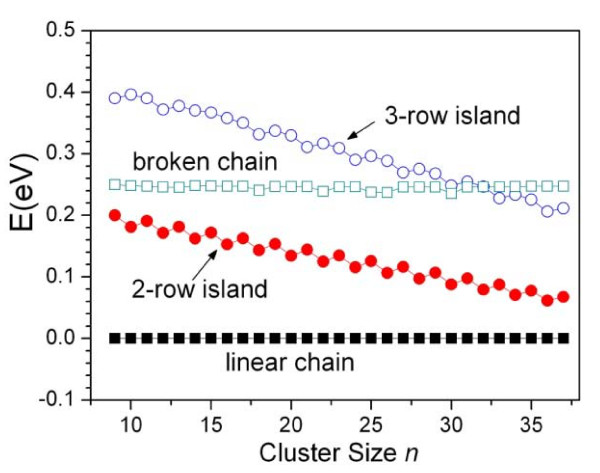
**The relative energy distribution**. Same as Figure 4, but for Ag(110) surface.

Therefore, according to Equation 11, we can predict the type change of the lowest-energy structure. Still take Cu(110) as an example (*ξ *= 5.26), from Equation 11, the change of the lowest-energy structure from one linear chain to two-row island will occur at $n=n_{1}^{c}=12$, and two-row island to three-row one at $n=n_{2}^{c}=\text{35}$, these predictions for the lowest-energy structure are in accordance with our GA optimization result. In Figure 4, we further give the relative energy distribution of the four structure types on Cu(110), the crossing of the lines means the change of the structure type, which just appears at the cluster sizes as Equation 11 given, i.e., at *n *= 12 and 35. On Ni(110) and Ag(110) surfaces, the $n_{1}^{c}$ and $n_{2}^{c}$ are also obtained from Equation 11 and given in Table 1, which are consistent with GA optimization results. On Ag(110) for example, *ξ *is 37.56 and then $n_{1}^{c}=\text{76}$ and $n_{2}^{c}=\text{227}$, which are much larger than those on Cu(110) (see Table 1). Accordingly, as shown in Figure 5, the type change of the lowest-energy structure is much slower than that on Cu(110) with the cluster size increasing.

**Table 1:** The energies of NN and effective NNN bonds and their ratio on metal surfaces

Surface	Cu (RGL)	Ni (EAM)	Ag (SEAM)	Pt (SEAM)
*E_nn_*	0.2404	0.2824	0.2569	0.3972
$E_{\text{nnn}}^{*}$	0.04570	0.04129	0.00684	−0.16753
*ξ*	5.26	6.84	37.56	−2.37
$n_{1}^{c}$	12	14	76	-
$n_{2}^{c}$	35	41	227	-
*A*	0.269	0.207	0.038	-

$n_{1}^{c}$ and $n_{2}^{c}$ are the critical sizes predicted by Equation 11, which are in accordance with those from GA optimization. *A *is the aspect ratio of the equilibrium island given by Equation 14.

Corresponding to the type change of the lowest-energy structure, the low-energy structures studied here show an interesting stepwise replacement in type with the cluster size increasing. For example, on Cu(110), there is only linear chain in the LES group for *n *≤ 5. At *n *= 6, the two-row island appears in the LES group. Our GA optimization shows that when the cluster size *n *increases, the energy of two-row island is increasingly lower than that of the linear chain, and at *n *= 12, as mentioned above, the two-row island becomes the lowest-energy structure of the cluster. When the size increases further, the linear chain gradually disappears from the LES group, meanwhile the three-row island appears. The two-row island maintains in the group. At *n *= 16, there is no linear chain in the LES group. When the cluster size becomes much larger than 16, similar to the case of linear chain, the energy of two-row island is increasingly higher than that of three-row island. At *n *= 35, as mentioned above, the three-row island becomes the lowest-energy structure. When cluster size increases further, the two-row islands are gradually excluded from the LES group, meanwhile the four-row island appears in the LES group. At that time, the three-row island maintains in the group. In one word, when the cluster size increases, the structures with more rows replace the ones with fewer rows step by step. The stepwise replacement of the low-energy structures also appears on Ag(110) and Ni(110) surfaces, the difference is only the speed of the replacement owing to the different ratio *ξ *and then $n_{R}^{c}$. For example, on Ag(110) surface, the speed of the replacement with the cluster size increasing is much slower than that on Cu(110) like Figures 4 and 5 for the change of the lowest-energy structure.

In terms of NN and NNN atom-atom interactions, we give a simple model Equation 7 to describe the energy of the cluster adsorbed on fcc(110) surface. In the above, we see that the model explains well the distinguishing features of the low-energy structures obtained by our GA optimization, including structure type varying with the surface species and cluster size, which suggest that the model is reasonable. The most important is that based on our model Equation 7, we can further explore the equilibrium shape of large islands on fcc(110) surfaces, which is difficult to be obtained directly by GA optimization owing to the heavy computation. For numbers of rows and lines in cluster, we have

(12) rl=n-d,

where *d *is the number of atoms needed for the cluster to form a complete *r *× *l *rectangular island, and it satisfies 0 ≤ *d *<*l*. For example, the structure (7) in Figure 1a has *d *= 3. When the cluster size *n *increases, the value of *d *linearly oscillates from 0 to (*l *− 1). Considering that the problem we are interested in here is the general shape of the low-energy islands in equilibrium state, we take the average value of *d *in Equation 12, i.e., *d *= *l*/2. Note that *r *and *l *need to have proper values to minimize the energy of cluster, i.e., minimize *Φ *Equation 8, and then with Equation 12 and *d *= *l*/2, we have:

$$r=\sqrt{\frac{n}{\xi }}-\frac{1}{2}$$

(13) $$l=\sqrt{n\xi }.$$

If the right side of Equation 13 is not an integer, then the close one which minimizes *Φ *is taken as the value for *r *or *l*. Then, we obtain the aspect ratio *A *of the equilibrium island:

(14) $$A=\frac{\left(r-1\right)\sqrt{2}a}{la}=\sqrt{2}\left(\sqrt{\frac{n}{\xi }}-\frac{3}{2}\right)∕\sqrt{n\xi }\approx \frac{\sqrt{2}}{\xi },$$

where *a *is distance between two nearest neighbor atoms. Note that we have used $\sqrt{n∕\xi }>>\text{3}∕\text{2}$ for large clusters and assumed that each NN bond has the same length in Equation 14. Therefore, the equilibrium shape of large cluster only relates with *ξ*, i.e., the ratio of NN and NNN bond energies. If the cluster has large *ξ*, the aspect ratio *A *is small, and then the equilibrium shape is long in [$1\bar{1}0$] direction and narrow in [001] direction. If the *ξ *is small, then the equilibrium shape with large aspect ratio *A *appears short and wide. For clusters on Ag(110), as shown in Table 1, our calculation shows that *A *is small, only 0.038. Such aspect ratio suggests the equilibrium shape of large clusters on Ag(110) is strip-like in [$1\bar{1}0$] direction, and it is consistent with the experimental observation in general [19].

**Figure 6:**
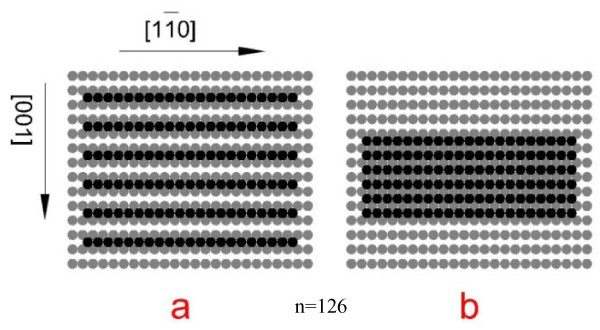
**Two types of structures for large adatom cluster on fcc(110) surface**. Loose (a) and compact (b) configurations of cluster n = 126.

From the distinguishing features of the structures in LES group and the simplified atomic interaction model Equation 7, we can further discuss the surface reconstruction qualitatively. On Pt(110), as mentioned above, there is only linear chain in the LES group, the reason is that the effective next nearest-neighbor atomic interaction in cluster is repulsive. The islands are all excluded from the LES group. The result suggests that the Pt adatoms on Pt(110) do not tend to form close-packed configuration but prefer the loose one which is continuous in [$1\bar{1}0$] direction but discontinuous in [001] direction, e.g., structure (a) in Figure 6, where two types of structures for large adatom cluster on fcc(110) are shown. The calculation shows that the energy of loose configuration as the structure (a) in Figure 6 is indeed much lower than that of the compact one as the structure (b) in Figure 6, and thus the former configuration has much higher frequency to occur than the latter one. When the cluster size increases, the compact configuration like the island (b) in Figure 6 forms the regular unreconstructed surface, while the loose configuration as structure (a) will form the surface with (1 × 2) reconstruction. Therefore, on Pt(110) surface, the (1 × 2) reconstruction has much higher frequency to occur than the regular unreconstructed arrangement. In other words, the (1 × 2) reconstruction would occur naturally on Pt(110), which in view of atomic interaction is caused by the repulsive NNN atomic interaction. According to the FIM observation, Pt(110) is indeed naturally form (1 × 2) reconstruction at room temperature [20].

For cluster on other surfaces, e.g., Cu(110) and Ag(110), different from the case on Pt(110), the compact configuration has much lower energy than the loose one because the effective next nearest-neighbor adatom-adatom interaction is attractive as mentioned above. Then, on Cu(110) and Ag(110) surfaces, the compact structure such as island (b) in Figure 6 has much higher frequency than structure (a). Therefore, contrary to Pt(110) surface, the Cu(110) and Ag(110) surfaces are unlikely to occur (1 × 2) reconstruction naturally, which are in good agreement with the observation of Zhang et al. [21]. These accordant results including the shape of large islands and the surface reconstruction reflect that our model Equation 7 really works although it is just based on the simplified two-body interaction.

## Conclusion

Groups of low-energy structures are obtained for clusters adsorbed on Ag(110), Ni(110), Cu(110), and Pt(110) surfaces by the genetic algorithm based on the EAM, SEAM, and tight-binding potentials. In order to explain or understand the low-energy structures, we give a model based on the simplified atom-atom interactions. The result shows that the difference of the low-energy structure on different surface is due to the effective NNN adatom-adatom interaction although it is very weak comparing to the NN atomic interaction. For a repulsive NNN atomic interaction, e.g., on Pt(110), there is only one type of structure in the LES group, i.e., linear chain. For an attractive NNN atomic interaction, e.g., on Ag(110), Ni(110), and Cu(110) surfaces, the structure type in the LES group is various, and when the cluster size increases the structure type with fewer rows will be gradually excluded from the LES group and replaced by the new one with more rows. The speed of replacement with the cluster size is determined by the ratio of the NN and NNN bond energies *ξ*. Based on our model, we also discuss the aspect ratio of the large island and the surface reconstruction on fcc(110) in the view of atomic interaction. It is shown that the aspect ratio is inversely proportional to *ξ*. On Ag(110) surface, for example, owing to large *ξ*, the equilibrium shape of the large island is strip-like in [$1\bar{1}0$] direction. The surface reconstruction is related to the NNN atomic interaction. On Pt(110) surface, the surface is likely to reconstruct naturally at room temperature because of the repulsive NNN atomic interaction. On other surfaces, e.g., Cu(110), however, owing to the attractive NNN atomic interaction, the natural surface reconstruction is unlikely to occur. These results are basically in accordance with the experimental observations.

## Competing interests

The authors declare that they have no competing interests.

## Authors' contributions

PZ, LXM, HZS, and JHZ wrote the computer program together, and PZ also performed the simulations and other calculations. WXZ, XJN, and JZ corrected the program and developed the algorithm. PZ and JZ give the model and explained the results. All the authors participated in the revision and approval of the manuscript.
